# Towards the implementation of large scale innovations in complex health care systems: views of managers and frontline personnel

**DOI:** 10.1186/s13104-016-2133-0

**Published:** 2016-06-28

**Authors:** Sonia Wutzke, Murray Benton, Raj Verma

**Affiliations:** The Australian Prevention Partnership Centre, Sydney, Australia; NSW Agency for Clinical Innovation, Sydney, Australia; Menzies Centre for Health Policy, University of Sydney, Sydney, Australia; Inca Consulting Pty Ltd, Sydney, Australia

**Keywords:** Keywords, Implementation, Health system change, Innovations

## Abstract

**Background:**

Increasingly, theorists and academic researchers develop, implement and test frameworks and strategies for improving the safety, effectiveness and efficiency of health care—at scale. The purpose of this research was to surface the views of health system managers and frontline personnel charged with implementing these improvement processes, to better understand key to success from personal experience.

**Results:**

A total of 17 out of 21 individuals invited to participate took part. Respondents, who were experienced senior managers and executives from various health agencies, provided comments via semi-structured discussions that lasted approximately 1 h. The discussions broadly focussed on what enables and inhibits the wider application of innovations to improve health service delivery. Respondents identified a number of broad factors that underpinned the successful and sustainable implementation of innovative initiatives: (1) a sound business case or ‘case for change’; (2) good preparation for the change process and thought given to how the initiative could be adapted to different contexts; (3) good engagement of clinicians, administrators and others; and (4) good support provided through the implementation phase, including having the right people, structures and strategies in place to coordinate implementation across the system.

**Conclusions:**

Measured responses that acknowledge both the tangible and less tangible aspects of a change process are required for the planning and implementation of large scale, successful and sustainable change initiatives across complex health systems.

## Background

Within a context of finite resources, it is difficult not to assent to concerns that the rate of growth in expenditure for health care is not sustainable. Internationally, health services are stretched by demands from population ageing, increasingly expensive technology, the growing prevalence of chronic diseases and high patient expectations [1–3]. Within this context, leaders, clinicians, professional bodies, government agencies and the community are calling for more efficient, safer, more value-adding healthcare [4].

Improving healthcare can be achieved through behaviour change at an individual practitioner level, with educational materials, conferences and courses, interactive small group meetings, academic detailing/educational outreach visits, use of opinion leaders, feedback on performance and reminders noted as being particularly influential strategies [5]. Further, small-scale improvements in healthcare are recognised in an abundance of published literature providing exemplars of improvements through local initiatives, typically carried out by a single health care organisation or service.

For optimisation of health system performance, however, it is argued that practitioner and local based interventions are insufficient alone. Rather, effective, efficient and sustainable health system improvement requires new, innovative, ‘at scale’ initiatives [6, 7]. Dubbed by some as ‘large-system transformation’ [8] it is argued that large-scale health system change can only be achieved by addressing the complex health system arena and its interplay of social factors, financing systems, organisational structures and processes, health technologies, and personal behaviours.

Increasingly, the literature reports a growing number of theories and frameworks for achieving large scale improvements across health systems—including the Centers for Disease Control and Prevention (CDC) Replicating Effective Programs [9], the Institute for Healthcare Improvement (IHI) Framework for Spread [10], the Quality Enhancement Research Initiative (QUERI) Framework [11], and the Veterans Affairs (VA) Systems Improvement Framework [12]—to name but a few. Further, several key factors have been identified as critical enablers of successful large-scale change across health systems, including: leadership [8, 13, 14]; being prepared for change [15]; ensuring engagement with individuals at all levels [8, 13]; having adequate resources and infrastructure in place [14, 15]; creating capacity amongst staff responsible for implementing the change [8, 15]; alignment of goals [14]; and establishing evaluation and feedback to support accountability and quality improvement. [8, 13–15].

In this busy landscape of frameworks, theories and key ‘ingredients’ for achieving successful and sustainable large-scale change in complex health systems, what remains unaddressed is how health system managers and frontline personnel have themselves personally experienced the implementation of innovations at scale. And from their ‘real world’ experiences what do they see as the keys to the success and sustainability of initiatives. The argument being that the complexities of health systems are such that published literature alone is insufficient for truly understanding cause and effect and solutions. Real world experience and insights from practice, both successes and failures, are essential adjuncts to the knowledge generated through theories and academic research.

The purpose of this research therefore was to:

1. surface the views of health system managers and frontline personnel charged with implementing large-scale, health system improvement initiatives, to better understand keys to success from their ‘real world’ experience
2. provide commentary on the confluence between the literature on health system improvements and the views of those working in practice to achieve these changes across health systems.

## Methods

The study methods followed published standards for undertaking and reporting qualitative research [16, 17]. Semi-structured interviews were undertaken with individuals invited to participate based on their likely ability to provide an informed contribution to the study, or to nominate other suitable candidates from their organisation. The initial contact list for invitees was agreed by the research team. The individuals invited were known to the research team to have had hands-on experience in the implementation of large-scale, health system improvement initiatives. Invitees reflected senior and middle managers of: the NSW Agency for Clinical Innovation [ACI, a statutory health organisation charged with designing and promoting improved healthcare across the State of New South Wales (NSW), Australia] [18]; the NSW Ministry of Health (responsible for the development, funding and implementation of health policy and monitoring of health system performance across the state of NSW) [19]; Local Health Districts (that operate public hospitals and provide health services to geographically defined communities); and innovation/redesign/improvement units from health departments of two additional States of Australia (Queensland and Victoria) and New Zealand.

A total of 21 potential participants were invited to participate through a personally addressed email from the Chief Executive of ACI. This was followed up by the research team within 1 week. To ensure the study captured informed perspectives all respondents needed to have relevant experience of at least 2 years. Further, all respondents were 18 years of age or over and able to provide informed written consent prior to participation.

Data collection consisted of individual, semi-structured interviews. Interviews were undertaken between July and August 2013 and where possible were face-to-face and audio-recorded.

Research questions, scope and methods were defined by the research team at an initial planning workshop, which was also attended by an independent consultant. The discussion guide followed a scenario of taking an innovation that has been shown to improve health outcomes and efficiency of health care delivery, and implementing it at a state-wide level. The research did not touch on the questions of how innovation occurs and what allows it to occur, but rather what enables and inhibits its wider application. The discussions also tended to be confined to scenarios where innovative systems or processes (and the change required to implement them) were promoted rather than mandated. More specifically, the discussion guide was structured to elicit views on: (1) What are the key mechanisms that influence and drive innovation and change in a health system, including sustainability of change? (2) What factors make a new initiative particularly vulnerable to failure if not addressed? and (3) Do mechanisms vary in effectiveness across issue and context?

Responses were anonymised and audiotapes reviewed by the interviewer (MB). Consistent with a grounded theory approach themes relevant to the aims of the research were generated from the content of the interviews rather than from a priori assumptions [20]. To ensure rigour and objectivity, the initial identification of themes and sub-themes was reviewed by a second researcher (SW). If there was inconsistency in interpretation a discussion was held to collectively agree and refine key themes. This process was also used to agree quotations reported in the results.

The research was approved by Sydney Local Health District Human Ethics Committee (Protocol No X13-0243, LNR/13/RPAH/302).

### Findings

Of the 21 individuals contacted, 17 (81 %) were interviewed. Interviews were approximately 1 h in duration and most were conducted face to face, with five conducted by telephone.

Informants identified a number of factors, which from their personal experiences underpinned the successful and sustainable implementation of innovations across complex health systems. These factors broadly covered four areas.

### Having a sound business case for change

Interviewees all agreed that successful innovation projects and processes were almost always underpinned by a strong ‘business case’ or a ‘case for change’. For these respondents, a number of attributes of a compelling case for change were identified, including:

- A *clear definition of the problem or need* with the extent quantified in the strongest possible terms.
- *Relevant contextual information* to illustrate root causes, exacerbating factors and the potential worsening of a problem.
- An analysis of the* cost of doing nothing* in terms of clinical outcomes, patient experience and cost to government.
- A *range of potential responses* or solutions for addressing the problem.
- Sound projections of the *resource implications and benefits* of introducing the proposed change, including return on investment for cost outlays. As one respondent said: *“You need to clearly state the case for change. There needs to be good costings”.*
- Professional *packaging*, to add credibility to the case. As one person said: *“Converting data into excellent information is the key”.*

### Being prepared for the change process, including adapting for different contexts

Almost all informants noted that there was no shortage of good ideas and sound cases for change that had not been taken any further, for one reason or another. For these respondents, the elements that underpin successful large-scale change included:

- Striking the *right balance between the fidelity of the ‘model’ and the way it is adapted to local context*. Informants thought that there was no ‘rule’ to be applied here other than to identify the core, ‘non-negotiable’ elements of the initiative and the areas where some modification is possible—a process of ‘flexible standardisation’. As one person said: “*You need to work out what things must be done, what should be done and what would be nice to do”.*
- Remaining *open to new ideas* and focusing on *outcomes* with the recognition that these can be achieved in more than one way
- Ensuring there is *local ownership* and establishing a *collaborative process* of refining and adapting the initiative to suit local environments. As one person said: *“Let the administrators decide who sits where. You can help them wrestle with it and make some suggestions but you can’t be too prescriptive”.*
- Rather than foisting ideas or new systems on people and organisations, ‘advertise’ the initiative (and the underlying need for it) and *support those who want to pursue it* rather than battle with those who are not interested. As one person said: *“The aim is to get them to grab it out of our hands—we need products that they want”.*
- Being ready for the almost serendipitous way in which some initiatives ‘fly’ and others do not. It is important to be *constantly on the look-out for opportunities* to coat-tail on other initiatives or to quickly position an initiative as a response to some emergent problem or ‘hot topic’.

### Promoting change through stakeholder engagement

A common theme expressed by almost all respondents was the importance of understanding that implementing any new initiative requires individuals to change, and sometimes, for organisational culture to change. In this space, many interviewees acknowledged the need for:

- An effective *engagement* strategy, from the outset, and particularly with the individuals with the power to influence change. As one person said: *“To get large scale change, you need to consider all and engage with all stakeholders*—*ask what do they need from us and what do we need from them?”* And a second said: *“change happens when there’s a marriage between clinical and administrative thinking”.*
- Clinicians to *champion* the change process. As one person said: *“Picking the right local champion is important…a person with trust and credibility”.*
- Clarity around personal *benefits*, so that people take an interest. As one person said: *“It’s about asking yourself ‘How do I get this project higher on your agenda?”*
- Acknowledging that *resistance* for change is inevitable and so it should be expected and managed. As one person said: *“You have to surface the resistance and then address it. If you’re not experiencing resistance, you just haven’t found it.”* And a second said: *“Look for the barriers, find the people who will resist—go see them and try to understand them”.*
- Encouraging people to think from a *systems perspective* rather than just thinking about their ‘own patch’ and recognising the implications of any change for others
- *Executive support* and overt commitment for the change. As one person said: *“It’s about getting someone in a position of power to care and then contracting with them”.*
- Sufficient *funding* to support the initiative
- *Change agents* who are credible and have the ability to influence. As one person said: *“You need good people who understand clinicians…people who have worked in the system…you need to be practical, grounded and able to talk to clinicians in their language.”* And a second said: *“It’s about personal relationships, interpersonal skills—getting people to champion ideas, to try to influence others”.*

### Ensuring good support throughout the implementation process

Respondents unanimously agreed that it is not enough to demonstrate a need, produce an effective solution and then get agreement to make change—there needs to be good implementation. Important factors in this space identified by respondents included:

- Good *project management* that takes a flexible rather than a mechanical approach. As one person said: *“It’s implementation, not installation”.*
- Taking an *incremental approach* where implementation is not rushed and the focus remains on outcomes not timelines.
- Providing good *support to the clinicians* who are championing the new way of doing things.
- Supporting ongoing *evaluation* to demonstrate effectiveness and efficiency in different contexts and provide feedback to help refine and improve the initiative.

## Discussion

There was significant clarity amongst respondents in this research as to what underpins successful and sustainable implementation of change across complex health systems and these views were consistent regardless of the seniority of the role the respondent held or the setting of their employment. Based on personal experiences, the broad factors seen to underpin the successful and sustainable implementation of innovations across a health system included: having a sound business case for the change; being prepared for the change process; promoting the change through good engagement of clinicians, administrators and others; and having the right structures and processes in place support implementation.

The views elicited through this research echoed many of the success factors identified in reviews in this space, including themes highlighting the need for: being prepared for change [15]; ensuring engagement with individuals at all levels with strong clinician and management support [14]; and having adequate resources and infrastructure in place to coordinate implementation [14, 15].

In addition, the views from respondents in this research also amplified the importance of having a sound and credible business case for the change as well as ongoing engagement and support throughout the implementation process. Respondents also highlighted the importance of local ownership and adaptation, whilst staying true to the agreed, core elements of the initiative. Finally, many respondents identified less tangible aspects they viewed as key to success, including: not forcing the change, but rather working with agencies in an incremental and locally relevant way to support the change; expecting and being prepared for resistance to the change; being ready to act on serendipitous opportunities; and having change agents with excellent project management skills and the experience and credibility to support and guide the change process.

A strength of this study was that views were included from a number of individuals experienced in the implementation of change initiatives across a large healthcare delivery system. Further, having an independent consultant conduct the interviews it was expected that bias from any existing relationships with interviewees would have been limited and there was no incentive to select results to fit a pre-determined position or agenda. Conducting semi-structured interviews ensured that discussions could be adapted to each interview, but this also meant that the research was unable to fully quantify the levels of agreement or contrast between respondents about issues that were raised by participants independently from the interview discussion guide. This study therefore does not allow for comparative analyses between respondents. Further, the discussions only lasted for a maximum of 1 h each and as such there was not the opportunity for any one respondent to convey everything they might have known about innovation and change in the health system. Further research is recommended that will allow deeper, real-world, case study analysis of implementation, both successful and unsuccessful, to better understand key enablers and barriers to positive and sustainable implementation of innovations across healthcare delivery systems. Finally, the focus in this research was on ‘across’ health system change, that is, views on the taking of an innovation and implementing it across a whole, state-based, healthcare delivery system (which includes many hospitals and health service delivery agencies). It could prove insightful to alter the lens of the enquiry to consider implications for ‘whole’ health system change, a subtle but important difference, that would examine whole of system components, for example the people, processes, activities, settings and structures, and the dynamic relationships between them [21], with the view that a better understanding of the system, its parts and whole, will enable better decisions about where and how to implement change.

## Conclusions

It is essential that health care delivery systems innovate at scale to optimise performance. Achieving successful and sustainable improvements across complex health systems is, however difficult. This research highlights the need for measured responses that acknowledge both the tangible and less tangible aspects for successful change. Combined, it is recommended that these elements be considered in the planning and implementation of large scale change initiatives across healthcare delivery systems.
